# Benefits from Immunization During the Vaccines for Children Program Era — United States, 1994–2013

**Published:** 2014-04-25

**Authors:** Cynthia G. Whitney, Fangjun Zhou, James Singleton, Anne Schuchat

**Affiliations:** 1 National Center for Immunization and Respiratory Diseases, CDC; 2 Immunization Services Division, National Center for Immunization and Respiratory Diseases, CDC

The Vaccines for Children (VFC) program was created by the Omnibus Budget Reconciliation Act of 1993 (1) and first implemented in 1994. VFC was designed to ensure that eligible children do not contract vaccine-preventable diseases because of inability to pay for vaccine and was created in response to a measles resurgence in the United States that resulted in approximately 55,000 cases reported during 1989–1991 (2). The resurgence was caused largely by widespread failure to vaccinate uninsured children at the recommended age of 12–15 months. To summarize the impact of the U.S. immunization program on the health of all children (both VFC-eligible and not VFC-eligible) who were born during the 20 years since VFC began, CDC used information on immunization coverage from the National Immunization Survey (NIS) and a previously published cost-benefit model to estimate illnesses, hospitalizations, and premature deaths prevented and costs saved by routine childhood vaccination during 1994–2013. Coverage for many childhood vaccine series was near or above 90% for much of the period. Modeling estimated that, among children born during 1994– 2013, vaccination will prevent an estimated 322 million illnesses, 21 million hospitalizations, and 732,000 deaths over the course of their lifetimes, at a net savings of $295 billion in direct costs and $1.38 trillion in total societal costs. With support from the VFC program, immunization has been a highly effective tool for improving the health of U.S. children.

Data from the 1980s suggested that measles outbreaks were linked to an ongoing reservoir of virus among high-density, low-income, inner-city populations (2). Although most children in these settings had a health-care provider, providers missed opportunities to give measles vaccine when children were in their offices, sometimes referring low-income children to another clinic where vaccines were available at no cost (3). Approximately 50% of children aged <19 years are eligible to receive vaccines through VFC (Immunization Services Division, National Center for Immunization and Respiratory Diseases, CDC, unpublished data, 2014).* Children can receive VFC-provided vaccine if they are Medicaid-eligible, uninsured, American Indian/Alaska Native, or, for underinsured children (i.e., whose health insurance does not fully cover immunizations), when they are receiving services at a federally qualified health center or rural health clinic (1). By providing vaccine for eligible children, at no charge, to public and private health-care providers who are enrolled in VFC, the program helped reinforce the “medical home.” Inclusion of specific vaccines in VFC is determined by recommendations of the Advisory Committee on Immunization Practices (ACIP).

To assess improvements in coverage during the VFC era, data were obtained from the United States Immunization Survey (USIS) for the period 1967–1985, the National Health Interview Survey (NHIS) for 1991–1993, and NIS for 1994–2012 (3,4). Children included in USIS and NHIS were aged 24–35 months and those in NIS were aged 19–35 months. USIS and NHIS data were from parental recollection of vaccines received, and NIS data were obtained through provider report.

The cost-benefit model for U.S. children born during 1994–2013 employed methods previously used for children born in 2009 (5). A decision analysis birth cohort model was constructed using data on immunization coverage; vaccine efficacies from published literature; historical data on incidence of illnesses, hospitalizations, and deaths from vaccine-preventable diseases before immunization was introduced; and recent vaccination period data (through 2013, if available; otherwise 2012 data were used for 2013) on these same disease outcomes. Vaccines included all those universally recommended for children aged ≤6 years except influenza vaccine, which has been modeled separately (6), and hepatitis A vaccine. Infants in hypothetical birth cohorts from the period 1994–2013 were followed from birth through death. Benefits of immunization included savings in direct and indirect costs that accrued from averting illnesses, hospitalizations, and deaths among the 20 birth cohorts. Program costs included vaccine, administration, vaccine adverse events, and parent travel and work time lost. Costs were adjusted to 2013 dollars, and future costs related to disease were discounted at 3% annually. The cost analysis was conducted from both health-care (direct) and societal (direct and indirect) perspectives, and net present value (net savings) was calculated.†

When the VFC program began in 1994, vaccines targeting nine diseases were provided: diphtheria, tetanus, pertussis, polio, *Haemophilus influenzae* type b disease, hepatitis B, measles, mumps, and rubella (Figure). During 1995–2013, five vaccines were added for children aged ≤6 years: varicella (1996), hepatitis A (1996–1999 for high-risk areas, 2006 for all states), pneumococcal disease (7-valent in 2000, 13-valent in 2010), influenza (ages 6–23 months in 2004 and ages 6–59 months in 2006), and rotavirus vaccine (2006). Since 1996, coverage with 1 dose of a measles-containing vaccine has exceeded *Healthy People*§ targets of 90%, up from <70% before the 1989–1991 outbreak (Figure). For other vaccines licensed before VFC, coverage also was higher in the VFC era, as measured by NIS, than in the pre-VFC era, as measured by USIS. In general, coverage for new vaccines introduced during the VFC era increased rapidly.

What is already known on this topic?

Vaccination is one of the most effective public health interventions. The Vaccines for Children (VFC) program was created by the Omnibus Budget Reconciliation Act of 1993 and implemented in 1994. VFC was created in response to low immunization coverage and the 1989–1991 measles outbreak in the United States.

What is added by this report?

In the 20 years since the VFC program was implemented, five new vaccines have been added to the routine infant immunization program, increasing the number of diseases prevented to 14. Vaccination coverage has remained near or above 90% for older vaccines. Because of vaccination, approximately 322 million illnesses, 21 million hospitalizations, and 732,000 premature deaths will be prevented among children born during this period, at a cost savings to society of $1.38 trillion.

What are the implications for public health practice?

The findings indicate the ongoing importance of maintaining and monitoring the U.S. immunization program.

Among 78.6 million children born during 1994–2013, routine childhood immunization was estimated to prevent 322 million illnesses (averaging 4.1 illnesses per child) and 21 million hospitalizations (0.27 per child) over the course of their lifetimes and avert 732,000 premature deaths from vaccine-preventable illnesses (Table). Illnesses prevented ranged from 3,000 for tetanus to >70 million for measles. The highest estimated cumulative numbers of hospitalizations and deaths that will be prevented were 8.9 million hospitalizations for measles and 507,000 deaths for diphtheria. The routine childhood vaccines introduced during the VFC era (excluding influenza and hepatitis A) together will prevent about 1.4 million hospitalizations and 56,300 deaths.

Vaccination will potentially avert $402 billion in direct costs and $1.5 trillion in societal costs because of illnesses prevented in these birth cohorts. After accounting for $107 billion and $121 billion in direct and societal costs of routine childhood immunization, respectively, the net present values (net savings) of routine childhood immunization from the payers’ and societal perspectives were $295 billion and $1.38 trillion, respectively.

## Discussion

This report shows the strength of the U.S. immunization program since VFC began; coverage with new vaccines increased rapidly after introduction, and coverage for older childhood vaccines remains near or above 90%. The ability of VFC to remove financial and logistical barriers hindering vaccination for low-income children likely played a significant role in obtaining high coverage. Successful delivery of vaccines to children of all income levels relies on participation of public and private health-care providers, insurance companies, state and federal public health officials, vaccine manufacturers, and parents. For pediatric health-care providers, VFC supported the “medical home” and reduced barriers to integrated, quality pediatric care with immunizations as the backbone of well-child visits. VFC also supports state-based immunization programs, which have transitioned from service delivery in public health clinics to quality assurance of private sector immunization and oversight of approximately 90 million VFC and other public sector doses distributed annually (Immunization Services Division, National Center for Immunization and Respiratory Diseases, CDC, unpublished data, 2013).

This analysis demonstrates the large number of illnesses, hospitalizations, and deaths prevented by childhood immunization. Because of sustained high coverage, many vaccine-preventable diseases are now uncommon in the United States. Measles was declared no longer endemic in the United States in 2000 (2), in contrast to model estimates that 71 million cases would have occurred in children born in the VFC era without immunization. Economic analysis for 2009 alone found that each dollar invested in vaccines and administration, on average, resulted in $3 in direct benefits and $10 in benefits when societal costs are included (5). Although the data presented here were generated with U.S. disease estimates and costs, the benefits are relevant to other countries where policymakers are considering return on investment in their immunization programs.

The model estimated more illnesses prevented by vaccination during the lifetimes of 20 birth cohorts than a report published in 2013 that found 26 million illnesses prevented in the U.S. population over the last decade (7) and a report published in 2007 that found prevention of 1 million to 2 million illnesses per year (8). These earlier assessments used disease reported through passive public health systems for baseline burden estimates, did not adjust for the increase in U.S. population over time, and assessed fewer vaccines than the model presented here, all factors that could explain their lower estimates.

The findings in this report are subject to at least three limitations. First, the benefits of hepatitis A vaccine, annual childhood influenza vaccine, and adolescent vaccines were not included. Second, the model did not account for all indirect vaccine effects on disease burden; for some vaccines, reduced transmission to unvaccinated populations has been a powerful driver of cost-effectiveness (9). Finally, for some diseases such as diphtheria, factors other than immunization might have contributed to lower disease risks in recent decades, and reductions resulting from these contributions have not been incorporated into the model; if such reductions were substantial, the model would overestimate the vaccine-preventable burden. However, a sensitivity analysis of the 2009 birth cohort model using the same methods suggested that, even with “worst case scenario” assumptions, early childhood immunization was cost-saving (5).

Although VFC has strengthened the U.S. immunization program, ongoing attention is needed to ensure that the program addresses challenges and incorporates methods that could improve delivery. Approximately 4 million children are born in the United States each year, each of whom is vulnerable to vaccine-preventable pathogens that continue to circulate. Importations from areas where measles is endemic are an ongoing challenge for public health workers and clinicians. Coverage with human papillomavirus vaccine for adolescent girls has not yet reached optimal levels. Essential program functions such as monitoring vaccine safety, coverage, and effectiveness and managing supply interruptions need ongoing attention, although the VFC stockpile has helped mitigate the impact of shortages (10). VFC, in conjunction with provisions of the Affordable Care Act that eliminate many co-payments for ACIP-recommended vaccines, minimizes financial barriers and thereby helps protect children from vaccine-preventable diseases.

## Figures and Tables

**FIGURE:**
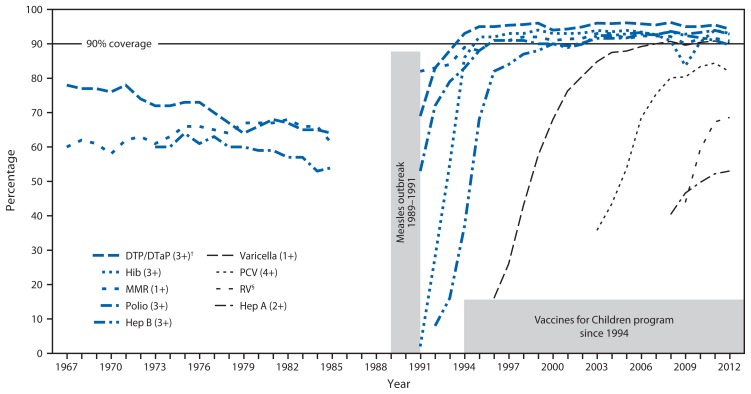
Vaccine coverage rates among preschool-aged children^*^ — United States, 1967–2012 **Abbreviations:** DTP/DTaP = diphtheria, tetanus, pertussis or diphtheria, tetanus, acellular pertussis; MMR = measles, mumps, and rubella; Hib = *Haemophilus influenzae* type b; Hep B = hepatitis B; PCV = pneumococcal conjugate vaccine; RV = rotavirus vaccine; Hep A = hepatitis A. **Sources:** United States Immunization Survey (1967–1985), National Health Interview Survey (1991–1993), and National Immunization Survey (1994–2012). No data are available for 1986–1990. ^*^ Children in the United States Immunization Survey and National Health Interview Survey were aged 24–35 months. Children in the National Immunization Survey were aged 19–35 months. ^†^ Numbers in parentheses refer to the number of doses of that vaccine being tracked in this figure. ^§^ For rotavirus vaccine, 2 or 3 doses are tracked, depending on the type of rotavirus vaccine received.

**TABLE:** Estimated number of illnesses, hospitalizations, and deaths prevented by routine childhood immunization for selected vaccine-preventable diseases among children born during the Vaccines for Children era — United States, 1994–2013

Vaccine-preventable disease*	Cases prevented (in thousands)		

	Illnesses	Hospitalizations	Deaths
Diphtheria	5,073	5,073	507.3
Tetanus	3	3	0.5
Pertussis	54,406	2,697	20.3
*Haemophilus influenzae* type B	361	334	13.7
Polio	1,244	530	14.8
Measles	70,748	8,877	57.3
Mumps	42,704	1,361	0.2
Rubella	36,540	134	0.3
Congenital rubella syndrome	12	17	1.3
Hepatitis B	4,007	623	59.7
Varicella	68,445	176	1.2
Pneumococcus-related diseases†	26,578	903	55.0
Rotavirus	11,968	327	0.1
**Total**	**322,089**	**21,055**	**731.7**

* Vaccines were considered as preventing disease for birth cohorts born in all years during 1994–2013 except for the following, which were only in use for part of the 20-year period: varicella, 1996–2013; 7-valent and 13-valent pneumococcal conjugate vaccines, 2001–2013; and rotavirus, 2007–2013.

† Includes invasive pneumococcal disease, otitis media, and pneumonia.

## Footnotes

* Additional information available at http://www.cdc.gov/vaccines/programs/vfc/awardees/program-management/surveys/pes-estimates.html.

† Additional information available at http://www.cdc.gov/vaccines/programs/vfc/pubs/methods/.

§ Additional information available at http://www.healthypeople.gov/2020/topicsobjectives2020/objectiveslist.aspx?topicId=23.
